# A Re-evaluation of the Anatomy of the Claustrum in Rodents and Primates—Analyzing the Effect of Pallial Expansion

**DOI:** 10.3389/fnana.2019.00034

**Published:** 2019-03-26

**Authors:** Daniel Binks, Charles Watson, Luis Puelles

**Affiliations:** ^1^School of Biological Sciences, The University of Western Australia, Perth, WA, Australia; ^2^The Perron Institute for Neurological and Translational Science, Perth, WA, Australia; ^3^Neuroscience Research Australia, Sydney, NSW, Australia; ^4^Department of Human Anatomy and IMIB-Arrixaca Institute, School of Medicine, University of Murcia, El Palmar, Spain

**Keywords:** claustrum, dorsal endopiriform nucleus, macaque, rodent, organization

## Abstract

The components of the claustrum have been identified by gene expression in mice, but there is still uncertainty about the location of homologous components in primates. To aid interpretation of homologous elements between rodents and primates, we used a current understanding of pallial topology, species-specific telencephalic deformation, and gene expression data. In both rodents and primates, pallial areas maintain conserved topological relationships regardless of relative differences in pallial expansion. The components of the claustrum in primates can, therefore, be identified on the basis of their conserved topological relationships and patterns of gene expression. In rodents, a fairly straight telencephalic long axis runs between the early septopreoptic and amygdalar poles of the pallium. In primates, however, the remarkable dorsal pallial expansion causes this axis to be distorted to form a C shape. This has resulted in a number of errors in the interpretation of the location of claustral components. These errors are likely to have resulted from the unexpected topographical positioning of claustral components due to the bent telencephalic axis. We argue that, once the telencephalic distortion has been accounted for, both rodents and primates have homologous claustral components, and that the topological relationships of these components are conserved regardless of differences in the relative expansion of pallial areas.

## Introduction

**Figure 1:**
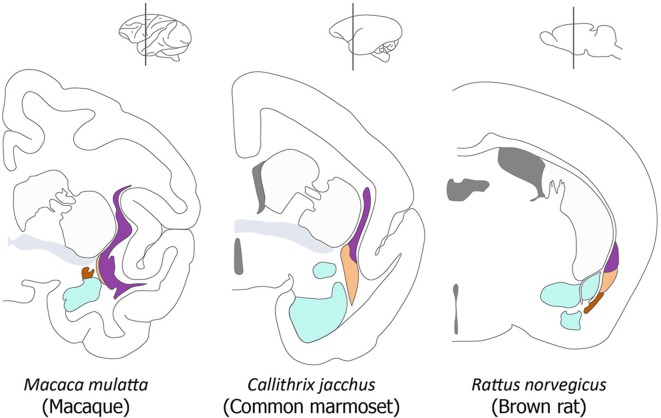
Organization of the claustrum in the macaque, marmoset and rat. A series of diagrams of coronal sections of primate and rodent brains to illustrate the commonly accepted view of the relative size and position of the components of the claustrum and their relationship to neighboring structures. In each diagram the principal claustrum is colored purple, the dorsal endopiriform nucleus (DEn) is colored beige, the ventral endopiriform nucleus is colored brown, the amygdala is colored cyan, the anterior commissure is colored gray, and the caudate-putamen and cortex are not colored. This diagram is adapted from Figure 1 in Smith et al. (2018).

In this study, we have attempted to identify the components of the claustrum in primate brains. While the elements that make up the claustrum have been confidently identified in the mouse (Puelles, 2014; Watson and Puelles, 2017), the situation in primates is complicated by distortion caused by the expansion of the dorsal pallium. The developing claustrum arises from a lateropallial sheet of cells that is found deep to the cortical layers of the mesocortical insular and perirhinal cortex. The insular/perirhinal mesocortex is transitional in layering complexity between olfactory allocortex (three layers) and isocortex (six layers). In primates, the mature claustrum is separated from the insular cortex by the extreme capsule, and it is separated from the white matter of the external capsule by a subjacent extension of the cortical subplate (Puelles, 2014). The current commonly accepted view of the organization of the claustrum is well illustrated in a diagram in a recent article by Smith et al. (2018). We have adapted their diagram in our Figure 1.

Along with subplate cells, the cells of the claustrum are the earliest born at the lateral pallium. Because of this they initially occupy a subpial mantle position, whereas later-born cells from the same lateropallial progenitors are destined to form the insular cortex. The insular cortex neurons migrate radially across the claustral primordium and reach the cortical surface, where they accumulate following the typical inside out mode of cortical plate stratification which is typical of all mammals. Superficial growth of the insular population thus gradually causes the claustrum to assume its final position deep to the insular cortex (Puelles, 2014). A number of migrations arise from the early subpial claustral anlage, as visualized with the use of the selective marker *Nr4a2 (Nurr1)* in labeling experiments (Puelles, 2017). Some claustral cells radiate tangentially into the depth of the ventropallial olfactory cortex, where they form the dorsal endopiriform nucleus, while others translocate dorsally into the overlying isocortex, where they contribute to the isocortical subplate population, or to a latexin-positive subpopulation described in layer5/6 (Puelles, 2014). Some other claustral cells penetrate radially into the insula (Puelles, 2014; Watson and Puelles, 2017).

However, these migration studies have been carried out in rodents, and there is still confusion about the identity of some of the claustral components in primates and other species on account of the extensive and differential (unequal) expansion of the surface of the dorsal pallium. One of the challenges in identifying claustral derivatives in primates is the C-shaped distortion caused by the differential areal expansion of the cortex, which results in the emergence of new frontal, occipital and temporal poles. Understanding the relationship between the invariant topologic position of the claustroinsular complex within the pallium and the merely apparent topographical claustrum displacement that accompanies pallial expansion in primates is the key to accurate comparative identification of claustral/insular components in different mammals.

## The Pallial Areas—A Topologically Consistent Bauplan

The concept of a topologically consistent morphological structure was highlighted in the classic 1922 work of D’Arcy Thompson (reprinted as Thompson, 1961). This work has particular relevance for the study of comparative neuroanatomy since it established that apparently unrelated adult biological forms can be generated by differential expression of a fundamental set of morphogenetic features. Nieuwenhuys and Puelles (2016) have argued that structures in the brain of vertebrates can be separated into an evolutionarily conserved framework of radial histogenetic units of the brain wall. These are produced from a topologically consistent molecular patterning process and they produce a corresponding map of molecularly-defined progenitor zones, as identified by combined codes of gene expression and analyses of reduced or excessive gene function. Homologous molecularly-defined structural units are observed in topologically consistent patterns in both amniotes and anamniotes, suggesting a highly conserved pattern of development equivalent to a bauplan (Puelles, 2001, 2011; Nieuwenhuys and Puelles, 2016). In anamniotes, the telencephalic pallium is divided into four topologically conserved sectors, identified as the medial pallium, dorsal pallium, lateral pallium and ventral pallium. These four pallial sectors exist as conserved field homologs in amniotes, irrespective of extensive evolutionary structural and functional variation (Puelles, 2014; Rodríguez-Moldes, 2009; Suryanarayana et al., 2017).

**Figure 2:**
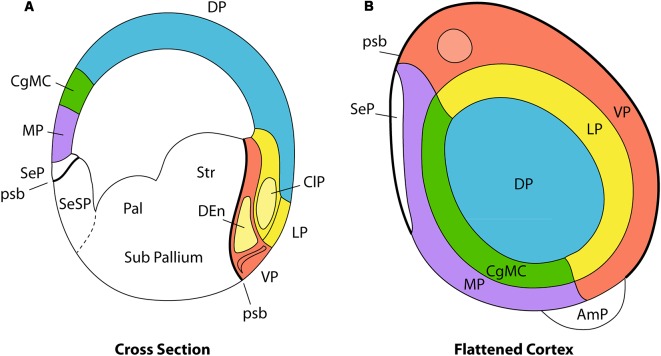
Pallial organization in amniotes. Panel **(A)** is a schematic transverse section through the developing telencephalon. The pallium is divided into the medial pallium (MP; violet), dorsal pallium (DP; blue), lateral pallium (LP; yellow), and ventral pallium (VP; red). Cingulate mesocortex (CgMC; green) lies intercalated between MP and DP. The light-yellow structure inside the red VP area represents the DEn, a population that migrates from the claustral anlage in the LP, whereas the red superficial corticoid formation is the olfactory cortex. The principal claustrum (ClP) is the light-yellow oval-shaped area found within LP, while the overlying yellow part of the cortical plate represents the insular cortex. Panel **(B)** is a planar flattened view of the pallial telencephalon, using the same color codes for the pallial sectors. Note that the circulate mesocortex is represented as closing medially the half ring of the LP, whereas the MP closes the half ring of the VP. The white domain rostromedial to the MP represents the rostral septal pallium domain (SeP, seen in **A** below the MP and above the psb). The pallial amygdala (AmP) representing the topologic caudal hemispheric pole lies outside the MP/VP ring. Note that the distinguished pallial sectors show clearcut topological relationships, both dorso-ventrally (**A**, cross section) and rostro-caudally (**B**, flattened cortex; septo-amygdalar axis). These relationships are kept invariant throughout morphogenesis irrespective of any deformation suffered by the long axis of the telencephalon. Abbreviations: pallial/subpallial boundary (psb), pallidum (Pal), striatum (Str), amygdalar pole (AmP), olfactory bulb (olb; light-red), pallial septum (SeP), subpallial septum (SeSP). Adapted from Puelles (2011).

Major evolutionary variations have emerged in the pallial bauplan of mammalian amniotes, including advanced inside-out layering of the dorsal pallium (isocortex), as well as a great areal expansion of the dorsal pallium observed in all mammals, particularly in primates (Puelles, 2011; Puelles et al., 2019; Figure 2). The topological relationships and underlying causal patterning mechanisms that emerged ancestrally to define the four fundamental pallial sectors are conserved throughout development. This means that topologic neighborhood relationships remain consistent even when structures are bent, stretched, twisted, or otherwise deformed during morphogenesis.

**Figure 3:**
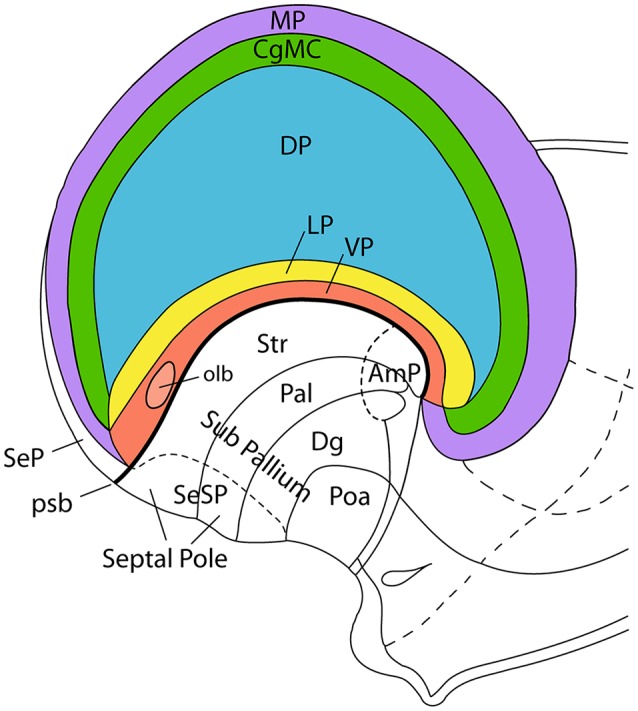
Incipient axial bending in the mammalian telencephalon, divided into pallial regions. This schema represents the pallial bauplan showing medial (MP; violet), dorsal (DP; blue), lateral (LP; yellow), and ventral (VP; red) pallial regions. The cingulate mesocortex (CgMC; green) is included as well, so that MP and VP form the external allopallial ring, while CgMC and LP form the transitional mesopallial ring that encloses the central DP island. The schema shows how the flat pallial map shown in Figure 1 can be visualized in a lateral view at an embryonic stage in which the septal and amygdalar poles of the hemisphere (Septal Pole; AmP) start to be pushed downwards by differential growth of the DP (isocortex). This incipient deformation carries with it the other components of the pallial map, which result similarly stretched into a curved inverted C-shaped arrangement. Each pallial region nevertheless maintains its fundamental dorso-ventral and rostro-caudal topology throughout the ensuing morphogenetic process. Abbreviations: pallial/subpallial boundary (psb), pallidum (Pal), striatum (Str), diagonal domain (Dg), preoptic area (Poa), pallial septum (SeP), subpallial septum (SeSP), olfactory bulb (olb). This figure was adapted from an illustration in Nieuwenhuys et al. (2008).

In addition to a consideration of dorso-ventral pallial relationships between the primary four pallial sectors (Figure 2A), we must also take into account the invariant rostro-caudal relationships (Figure 2B). It seems that the septo-preoptic region can be considered to be the rostral pole of the developing telencephalon, while the amygdalar region may represent its caudal pole (Nieuwenhuys and Puelles, 2016; Figure 2B, but compare Figure 3). Note that in this respect we are not referring to the long axis of the whole forebrain, but just to a subsidiary long axis of the evaginated telencephalic territory, which is the dissected telencephalic vesicle.

**Figure 4:**
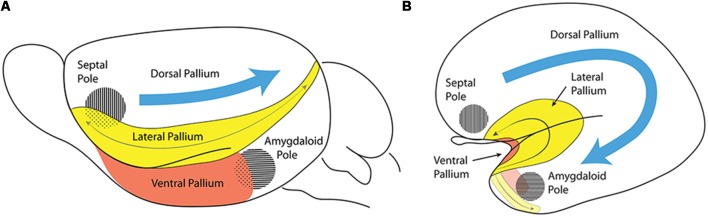
Comparison of the lateral pallium (LP) long axis in a rodent **(A)** and a primate **(B)**. These schemata represent the invariant position of the insular/perirhinal mesocortex (LP) relative to the olfactory cortex (ventral pallium, VP) and the primary septal and amygdalar poles of the hemisphere in a rodent **(A)** and in a primate **(B)**. The length axis of the rodent LP enclosing the claustroinsular complex is relatively undistorted (gray arrow, **A**), whereas the length axis of the primate LP undergoes a complex deformation. The frontal tip of LP points into the posterior orbital region of the frontal lobe, its middle part gets bent upon itself to form the insular cortex, and its caudal tip sharply bends around the medial aspect of the temporal pole (gray arrow, **B**). Note the VP (red) can be easily distinguished underneath the LP (yellow) in rodents **(A)**, while its topologically invariant position is found in the primate along the insular limen and ending caudally next to the uncal amygdalar complex. Consistently with the deformation suffered by the length axis of LP and VP as the temporal lobe emerges, the conserved topologic dorsoventral dimension now points from the insula into the insular limen. By using the septal and amygdalar poles as reference points, it is evident that the LP, which is dorsal to the amygdaloid pole in a rodent, acquires a quite different relative topography in the temporal lobe in primates. In primates, the long axes of both the LP and VP start flat rostrally, approximately on the horizontal plane, but becomes oriented vertically as the axis curves under the temporal pole and reaches the underside of the temporal cortex.

Pallial growth during the diverse stages of mammalian development, including the postnatal period, affects the four primary pallial sectors in different ways. Less expansion is manifest at the olfactory ventral pallium, the claustroinsular lateral pallium, and the hippocampal medial pallium, while the largest surface growth clearly occurs both ontogenetically and phylogenetically at the dorsal pallium. The dorsal pallium (isocortex) undergoes massive and differential areal expansion in many mammalian species, leading to an evolutionary late protrusion of novel frontal, occipital and temporal poles. The intermediate area occupied by the insular cortex shows limited surface expansion because it is physically anchored to the neighboring ganglionic eminences, which constitute an adjacent histogenetic unit whose neural wall grows in thickness. This combination results in an anteroposterior axial bend of the massively expanding parts of isocortex relative to the insula (eventually covered by opercula) and the adjacent ganglionic eminences. This axial C-shaped bend pushes the primitive amygdalar (caudal hemispheric reference point) passively into the uncal end of the temporal lobe, stretching and bending in the process the intercalated claustroinsular lateral pallial complex (Figure 4). These deformations vary in magnitude in different mammals, but they do not change the conserved relationships existing *ab initio* between the four pallial sectors, even if their respective molecularly-defined limits become difficult to visualize anatomically. In any case, the primary septo-preoptic and amygdalar telencephalic poles can be used as homologous topologic rostral and caudal reference points towards interpreting axial orientation (Figure 4).

The septo-amygdaloid axial bend, or C-shaped curve, can be clearly observed in primates, where the amygdala, representing the caudal telencephalic pole, is eventually found midway between the topographically “rostral” and “caudal” poles of the mature cerebrum (Swanson, 2018). Note that in Swanson’s flat map (his Figure 3), no topologic representation is pretended, since an arbitrarily straight fronto-occipital topographic axis is implied, and therefore no morphogenetic bending of the hemisphere is accounted for. On the other hand, our Figure 4 pictures the way in which the relatively small ventral pallium maintains its original topological relationships with both the septal region and the amygdalar region, whilst the dorsal pallium undergoes massive expansion around these points. Understanding the significance of the way in which the amygdalar area forms the true caudal reference point is particularly important in primates, as the occipital cortex falsely appears to form the caudal pole of the cerebrum. The massive growth of the occipital cortex causes it to overhang (topographically) the cerebellum in primates.

## The Concept of the Lateral Pallium

The concept of the lateral pallium has recently been updated by Puelles (Puelles, 2014, 2017; Watson and Puelles, 2017). The recognition of the lateral pallium as a distinct pallial sector clarifies its relationships with adjacent dorsal and ventral pallial sectors (Figure 2A). The lateral pallium forms part of the ring of mesocortical structures that surround the isocortex, as summarized by Puelles et al. (2019). Puelles (2014) argues the caudal end of the mesocortex does not converge into the amygdala, but continues between the temporo-occipital and the entorhinal cortical regions as the perirhinal/postrhinal mesocortex. In their turn, these caudal mesocortical regions are continuous with the posterior cingulate cortex in the medial wall of the hemisphere [lateral pallium (LP), Cingulate mesocortex (CgMC); Figures 2B, 3]. Similarly, the rostral insular mesocortex connects with the anterior cingulate cortex *via* the posterior orbital area (Figures 2B, 3). Outside this mesocortical ring is the allocortical ring, formed by olfactory ventral pallium and hippocampal medial pallium. The olfactory ventral pallium extends *via* the entorhinal cortex into the hippocampal medial pallium, and the supracommissural and precommissural hippocampus reaches rostromedially the olfactory bulb, and so closes this outer allocortical ring (Figures 2B, 3). The mesocortical and allocortical rings both participate in the C-shaped deformation of the pallium (Figures 3, 4), but it is important to note that the amygdala lies outside of the two rings (Puelles et al., 2019; Figure 3). An appreciation of these relationships allows structures such as the claustrum to be investigated in light of conserved topology in the same way as other pallial structures. In the case of primates, the claustrum often adopts a triangular shape, with an apparent rod-like extension into the posterior cortex. Due to the topographic appearance of occipital cortex forming the new “posterior” pole of the mature neocortex, this rod-like claustral extension can easily be misinterpreted to be the *caudal* pole of the claustrum, leading to further errors in interpretation. One such error is the attribution of the dorsal endopiriform nucleus to ventral aspects of the principal claustrum along the entire rostro-caudal topographic axis, instead of its tangentially migrated location deep to the ventropallial piriform cortex.

In primates, the reduced ventral pallium is restricted to the olfactory bulb, insular limen, and olfactory parts of the temporal uncus. The lateropallial insula is also much deformed compared with that seen in rodents. Given this deformed arrangement of the cortex in primates, we would expect to see a marked bend in the topologic long axis of the claustral-insular complex in order to maintain the integrity of the rostro-caudal topological relationships of the allocortical and mesocortical rings around the deformed dorsal pallium. Note that the perirhinal cortex of primates appears within the entorhinal sulcus at the rostromedial aspect of the temporal lobe, and the postrhinal cortex is represented by the parahippocampal area that connects with the retrosplenial cingulate region.

## Support for the Concept of a Change in the Long Axis of the Claustrum

Combining an understanding of the bent topologic long axis of the lateral pallium (and therefore of the claustrum) with our current understanding of its overall topology may help to provide new insights to its organization, structure and function. Afferent and efferent projections of the claustrum have been studied using a range of techniques, including cortical ablations and anterograde and retrograde axonal transport (Druga, 2014). These studies, along with electrophysiological investigations, have shown the presence of specific subregions in the claustrum across a range of species, including primates and rodents (Sherk, 2014). Such organized subregions have always been described as being aligned with the rostro-caudal topographic long axis in rodents, and also are reported to vary along the topographic long axis of the claustrum in primates (but they have not been related to the bent topologic axis underlined in Figure 4B). The presence of a bent long axis is implicit in a study of principal claustral projections to the prelunate gyrus (Baizer et al., 1997), where sagittal sections clearly illustrate the presence of a curved field of retrogradely labeled cells that coincides with our postulated bent axis. A bent long axis can also be distinguished in the temporal claustral expansion of the cat, where claustro-cortical projection fields appear to run first rostro-caudally and then dorso-ventrally as we move from the anterior portion of the claustrum to the caudal claustrum (Beneyto and Prieto, 2001). Further support for this concept is found in a study by Baizer (2001), in which she examined the serotonergic innervation of the primate claustrum. In the rostral claustrum, serotonergic axons were seen to run predominantly dorso-ventrally, yet in the ventral portion of the caudal claustrum there appeared many puncta and the axons were mostly short and randomly oriented, suggesting the axons might be running rostro-caudally at this locus (but maintaining orthogonality with the bent topologic axis).

However, these studies need to be interpreted with care, since not all of the results are consistent with the concept of a bent long axis. A study by Pearson et al. (1982) suggested that the claustrocortical connective fields may point toward the dorso-caudal aspect of the claustrum. As noted above, old assumptions concerning the dorsal endopiriform nuclues (based on studies which were unaware of its migrated nature) may have led to the dorsal endopiriform nucleus being identified as the ventro-caudal part of the principal claustrum. This error may have resulted in relevant data in these studies being overlooked. This is further complicated by the distorted shape of the claustrum, which makes it difficult to produce diagrams that adequately represent its morphology.

It is worth noting that large pools of claustral cells, referred to as puddles, aggregate in varying locations of the claustrum in a variety of mammals (Johnson et al., 2014). Further evidence for bending in the claustral long axis is also present in these puddles. For example, in the red fox a dorsally located cell pool remains peripheral (i.e., topologically dorsal) as the claustrum enters the temporal lobe (Johnson et al., 2014; see their Figure 1).

## Next Steps in the Investigation of Developmental Mechanisms in the Claustrum

While it is important to examine the morphological constraints and axial changes operating in principal claustral evolution, we must also consider whether the axis shift and the consequent change in claustrum morphology actually represent a meaningful change in the internal organization of the structure, or in the organization of its afferent/efferent connections. Little is known about the factors underlying the establishment of claustro-cortical topography, and no clear mechanisms have been proposed for its organization. These mechanisms could include chemical organization, neuronal activity, axonal sorting, and temporal ordering (Imai and Sakano, 2011). For example, changes in surface position and temporal order of developing axonal connections have been shown to influence sensory maps (Murphey et al., 1980). Mechanical cues can also play a role in organizing topography, where differential tension can affect axonal pathfinding (Gangatharan et al., 2018). Mechanical cues related to axial bending could conceivably play a role in the generation of different patterns of connectivity in the context of variation in cortical expansion. Thus, the bent topologic long axis of the primate claustrum is probably not responsible for an intrinsic shift in its structural organization, but rather the change in claustral morphology may affect the topographic organization of its connections. These concepts are highly speculative and require further investigation.

## Definition of Individual Claustral Elements in Rodents

Given that the rodent claustrum does not undergo a sizeable axial deformation, such as seen in primates (Figure 4A), we have searched for homologies by comparing the current understanding of rodent claustral organization with the predicted topographical deformation in primates.

A number of markers have been used to identify subdivisions of the claustrum. The anatomy of the rodent claustrum was studied with the Golgi technique by Ramón y Cajal (1900), and he correctly concluded that the claustrum was a distinct structure that was neither cortical nor striatal. Brodmann (1909) originally divided the claustrum into dorsal and ventral parts, but Loo (1930) later renamed the two parts as the principal claustrum and the dorsal endopiriform nucleus respectively. In rodents and other species lacking an extreme capsule, identifying the claustrum in Nissl stained sections is challenging. However, the advent of histochemical, immunohistochemical, and gene expression markers now makes it possible to distinguish the claustrum from the adjacent cortical layers of the insular cortex. The expression of genes such as *Nr4a2 (Nurr1), Latexin, Cux2* and* NetrinG2* has proven useful in identifying the principal claustrum as distinct from the dorsal endopiriform nucleus (Puelles, 2014; Watakabe et al., 2014). In contemporary brain atlases, the principal claustrum in rodents (leaving aside the dorsal endopiriform nucleus) has been further divided into dorsal and ventral portions, based on differential acetylcholinesterase (AChE) staining in rats (Paxinos and Watson, 2007), and in mice (Paxinos and Franklin, 2013), and this convention was adopted by Puelles (2014). Parvalbumin and calbindin (markers which are thought to label tangentially migrated subpallial inhibitory neurons) have also been used to define the subnuclei of the claustrum in the rat (Druga et al., 1993) and mouse (Real et al., 2003). Parvalbumin has proven to be a particularly useful marker for defining the ventral claustrum because it shows almost no staining in the dorsal endopiriform nucleus or dorsal claustrum in rodents (Paxinos et al., 2009). Other markers, such as *Ctgf*, have also been used in an attempt to selectively identify the dorsal endopiriform and claustral subplate, though this marker essentially labels the subplate component of the claustral complex in rodents (Wang et al., 2011; Watson and Puelles, 2017).

Whilst Paxinos and Watson (2007) and Puelles (2014) have adopted a separation of principal claustrum into dorsal claustrum and ventral claustrum, it has also been suggested that the principal claustrum can be broken into core and shell regions, based on the expression of *Gng2, Crym*, and calcium-binding proteins (Dávila et al., 2005; Mathur et al., 2009). These core/shell subdivisions largely seem to correspond to the principal and subplate claustrum components of Puelles (2014).

## Comparing Claustral Markers in Rodents and Primates

Studies comparing markers between rodents and primates show remarkable homogeneity. The expression of *Latexin, Nr4a2 (Nurr1), Cux2, NetrinG2*, co-expression of *Cux2* and *Nurr1*, and co-expression of *NetrinG2* and *Nurr1* all reveal close similarities between rodents and primates (Watakabe et al., 2014). Mathur et al. (2009) also provided evidence of a claustral core and shell in primates; they showed that the *Crym-*positive shell (the claustral subplate) is most clearly seen in the ventral portion of the principal claustrum.

However, some other claustral markers do reveal differences between rodents and primates. In rodents, AChE is absent from the ventral claustrum but prominently labels the dorsal claustrum (Paxinos and Watson, 2007). In the marmoset atlases of Paxinos et al. (2012) and Hardman and Ashwell (2012), AChE staining appears to be of little value in distinguishing subnuclei because the background staining is uniform across the entire principal claustrum. However, in the rhesus monkey, AChE staining clearly outlines different claustral elements (Paxinos et al., 2000), even though the atlas labeling does not always reflect the subdivisions we have identified in this article. The distribution of calcium-binding proteins does not support the existence of a distinct dorsal claustral homolog in primates, because this staining is uniform throughout the principal claustrum (Reynhout and Baizer, 1999). It also appears that some markers do not target the same neuron types in different species. For example, parvalbumin appears to stain projection neurons in the monkey, and these neurons are presumably glutamatergic and excitatory. However, parvalbumin stains what are presumably inhibitory local circuit neurons in the rat (Reynhout and Baizer, 1999). However, such differences may not be truly reflective of differences in the ontogenetic origin or anatomical organization of cell populations, as the specificity of calcium-binding proteins in homologous structures may vary from species to species (Baimbridge et al., 1992).

**Figure 5:**
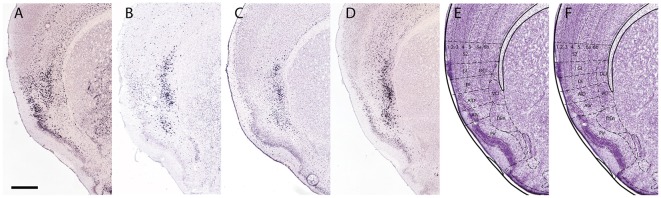
The claustrum shows core and shell expression in mice. This figure shows the expression of *Crym* **(A)**, *Synpr* **(B)**, *Nr4a2* **(C)** and* Ntng2* **(D)** in the mouse claustrum. *Crym* is negative in the dorsal endopiriform nucleus (DEn) but it surrounds the expression of *Synpr, Nr4a2* and *Ntng2* in the ventral claustrum (VCl). The core and shell is less prominent in the dorsal claustrum (DCl), with the expression of all four markers appearing more evenly distributed. Panel **(E)** shows the current definition of the DCl, VCl, and DEn based on their representation in the mouse brain atlas of Paxinos and Franklin (2013). Panel **(F)** shows suggested revisions to the definition of the DCl, VCl, DEn and insular region. The VCl corresponds with the agranular regions, the DCl corresponds with the dysgranular (DI) and granular (GI) insula regions and the DEn corresponds with the piriform cortex (Pir). The area given to the DCl should be expanded to include the complete dorso-lateral expression of *Synpr, Nr4a2* and *Ntng2*. Secondary somatosensory cortex (S2), piriform cortex (Pir), agranular insular cortex dorsal (AID), agranular insular cortex ventral (AIV). Scale bar - 500 μm. Images **(A–D)** © 2010 Allen Institute for Brain Science. Allen Mouse Brain Atlas (Lein et al., 2007). Available online at: mouse.brain-map.org

**Figure 6:**
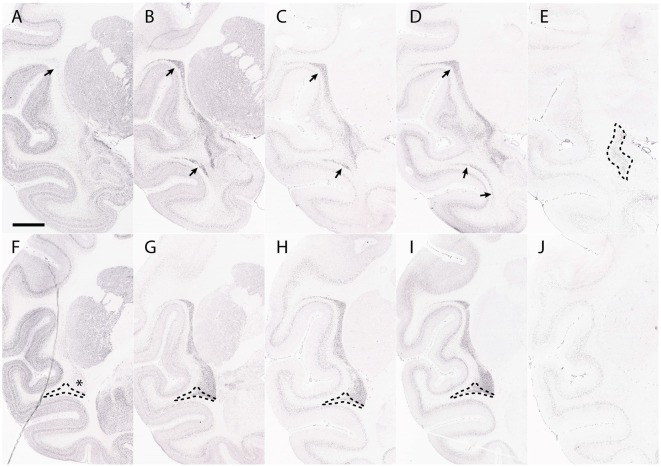
The claustrum shows core and shell expression in the macaque. This figure shows the expression of *Crym* **(A,F)**, *Synpr* **(B,G)**, *Nr4a2* **(C,H)**, *Ntng2* **(D,I)** and *Ctgf* **(E,J)** in the macaque claustrum. Panels **(A–E)** represent sections taken at the level of anterior temporal cortex. Panels **(F–J)** represent sections taken caudal to the dorsal endopiriform nucleus (DEn). *Crym* expression forms a shell at the edges of *Synpr, Nr4a2* and *Ntng2* in the ventral claustrum (VCl) (asterisk, **F**). The upper portion of the principal claustrum (ClP) has a more even distribution of *Crym, Synpr, Nr4a2* and *Ntng2*, which is homologous with the dorsal claustrum (DCl) in rodents (upper arrows, **A–D**). There is a lower portion of the ClP that has weaker *Synpr, Nr4a2*, and *Ntng2* expression, appears outside the “shell” region of the VCl and has weak mixed *Crym* expression (outlined region, **F–I**). These criteria, alongside the topological argument, identify this region as a continuation of the DCl traveling around and under the temporal cortex (lower arrows, **B–D**; outlined region, **F–I**). Panel **(E)** shows a selective expression of *Ctgf* in the DEn. The DEn appears directly caudal to the piriform cortex and disappears in further caudal sections **(J)**. Scale bar - 4,200 μm. Images **(A–J)** © 2009 Allen Institute for Brain Science. NIH Blueprint Non-Human Primate (NHP) Atlas (2009). Available online at: blueprintnhpatlas.org.

Despite these differences, we suggest that the claustrum can be divided into two distinct homologous regions in both rodents and primates. In rodents, the presence of distinct dorsal and ventral principal claustrum parts was demonstrated by the genetic markers used by Johnson et al. (2014) and Puelles (2014). The dorsal principal claustrum appears to underlie the granular/dysgranular insular cortex and the ventral principal claustrum appears to underly the agranular insular cortex. In rodents, the ventral claustrum can be divided into shell and core regions, based on the expression of *Nr4a2, Ntng2* and *Synpr* in the core and the expression of *Crym* in the shell (Figure 5). The pattern in dorsal claustrum is more homogeneous, with more *Crym* expression appearing deep to and mixed with the* Nr4a2, Ntng2* and *Synpr* core structure. A similar situation is observed in the primate principal claustrum, where the ventral claustrum shows a clear core and shell structure based on *Nr4a2, Ntng2*, *Synpr* and *Crym* differential expression (Figure 6). The dorsal claustrum instead shows mixed expression of *Nr4a2, Ntng2*, *Synpr* and *Crym*, which is not dissimilar to the mixed expression seen in the dorsal claustrum of rodents. This implies that the boundary of the dorsal claustrum in rodents should be expanded to include the dorso-lateral expression of *Nr4a2, Ntng2*, *Synpr* and *Crym* (Figure 5F). These expression patterns suggest that the dorsal claustrum organization in rodents and primates is basically similar.

The identification of the boundaries of the dorsal endopiriform nucleus in primates appears to have suffered from a misinterpretation of the long axis of the claustrum and insula. Many past anatomical attributions appear to have been based on an incorrect assumption that the dorsal endopiriform nucleus, as seen in coronal sections, is simply the ventral part of the principal claustrum, rather than a tangentially migrated claustral cell population developing ectopically within the ventral pallium (Puelles, 2014). This error is evident in the description of the macaque claustrum areas by Kowiański et al. (1999); who claim that in this monkey the dorsal endopiriform nucleus runs along the entire ventral length of the claustrum, as is found in rodents (Figure 3). The same error is also apparent in the marmoset atlas of Paxinos et al. (2012), where the dorsal endopiriform nucleus is identified under the entire ventral border of the claustrum. It is likely that these errors occurred because the authors did not take into account the much more elaborate axial bending of the rostro-caudal axis (topologic long axis) in primates. In contrast, Johnson et al. (2014) showed that a combination of *Ctgf* and *Rorb* expression identifies the dorsal endopiriform nucleus in both rodents and primates. They found that these labels are only seen within the ventropallial region immediately deep to the piriform cortex (Figure 6E), but not in more caudal sections (Figure 6J). This expression pattern is consistent with the predicted position of the dorsal endopiriform nucleus based on topology.

**Figure 7:**
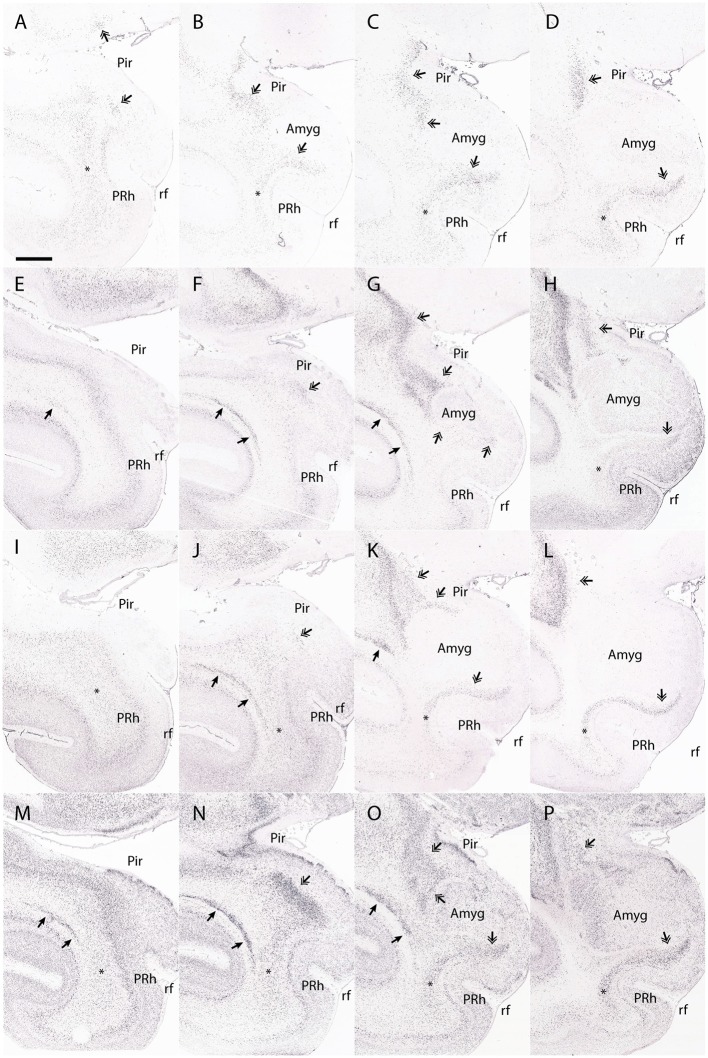
The dorsal endopiriform nucleus (DEn) in the macaque. This figure shows the expression of *Ctgf* (**A–D)**, *Ntng2* **(E–H)**, *Nr4a2* **(I–L)**, and *Synpr* **(M–P)** in the macaque DEn. Note that a coronal section through the temporal cortex may contain entorhinal cortex at the medial surface next to the rhinal fissure, yet may contain periamygdaloid olfactory cortex in a more dorsal location, making it difficult to accurately identify the transition between each area. *Ctgf* expression is seen in the DEn deep to the piriform cortex (**A–D**, double arrowhead). The DEn has a caudal part that is topologically inferior to the perirhinal cortex (in this case, medial to the perirhinal cortex) and continues until the periamygdaloid olfactory cortex transitions into the entorhinal cortex (**D**, double arrowhead). However, while *Ctgf* is useful for identifying the DEn, it also labels the subplate, and so other claustral markers must be interrogated to accurately separate the dorsal endopiriform and subplate from the claustral components. For example, *Ntng2* expression strongly labels the principal claustrum (**E–G**, single arrowhead), although only weak expression is seen in the topologically caudal claustrum that is found deep of the perirhinal cortex (**H**, asterisk). The DEn is also strongly labeled (**E–H**, double arrowhead). *Nr4a2* expression clearly shows the caudal aspects of the dorsal claustrum that lie deep to the perirhinal cortex (**I–L**, asterisk). The DEn is also labeled (**J–L**, double arrowheads), and maintains a topologically accurate location, deep to the piriform cortex and periamygdaloid olfactory cortex. *Synpr* expression is present in the DEn (**M–P**, double arrowhead), particularly the topologically caudal aspects that lie deep to the periamygdaloid olfactory cortex (**P**, double arrowhead). Symbols and abbreviations: Amyg—amygdala; Pir—piriform cortex; PRh, perirhinal cortex; rf—rhinal fissure; single arrowhead—dorsal claustrum; double arrowhead—dorsal endopiriform nucleus; asterisk—topologically caudal claustrum. Scale bar - 8,400 μm. Images **(A–P)** © 2009 Allen Institute for Brain Science. NIH Blueprint NHP Atlas (2009). Available online at: blueprintnhpatlas.org.

A further issue to be considered is the periamygdaloid claustral formation that Puelles (2014) renamed as the caudal endopiriform nucleus. He interprets the caudal endopiriform nucleus to be a derivative of the claustral population that lay deep to the perirhinal insular cortex early in development. This cell group apparently translocates secondarily *en masse* to lie deep to the periamygdaloid olfactory cortex, leaving practically no claustral remnant at the original position (Puelles et al., 2019). There are currently no markers that allow the main part of the dorsal endopiriform nucleus to be distinguished from its caudal counterpart that lies over the periamygdaloid olfactory cortex, so that the caudal part can only be distinguished by its relationship to the overlying cortex. Likewise, the perirhinal cortex differs from the typical insular cortex in that it does not overlie an obvious claustral element. However, the perirhinal cortex lies superficial to a migrated population that is related to the dorsal endopiriform nucleus. The dorsal endopiriform nucleus thus appears to be continuous across its endopiriform and periamygdaliod endopiriform components in rodents. The dorsal endopiriform nucleus also appears continuous across both the piriform cortex and the periamygdaloid olfactory cortex in the macaque, and finally ends in the same topological position (ventral in the rodent, dorsomedial in the macaque) to the topologically caudal aspect of the claustrum proper (Figure 7). Given the limitations of coronal sections when studying the dorsal endopiriform nucleus and other claustral derivatives in primates, as well as the lack of a distinct caudal endopiriform nucleus marker, we are unable to comment on the transition between the dorsal endopiriform nucleus and the potential existence of a distinct topologically caudal endopiriform nucleus.

## Problems With Interpretation of Components of Claustrum and DEn

The misattribution of the dorsal endopiriform nucleus in primates highlights the problem with naming structures based on their appearance within a single plane of section, without keeping in mind topographic displacing effects of axial bending that occurred during ontogenesis. In rodents, the dorsal endopiriform nucleus lies deep to the piriform cortex, ventral to the claustroinsular complex; it thus sits in a dorsal position within the endopiriform region in a coronal section (Figure 1). However, in the macaque, the majority of the piriform cortex is found on the insular limen and the antero-medial uncal surface of the temporal lobe (Figure 3), due to the bent cortical axis and resulting deformation. Whilst the dorsal endopiriform nucleus is still correctly identified immediately deep to the piriform cortex, it appears topographically caudal, and not dorsal, within the endopiriform region. It should only be identified on the basis of its relationship with the piriform cortex, and not simply because it lies ventral to the claustrum.

As noted above, the use of “dorsal” or “ventral” to describe nuclei creates difficulties when attempting to search for homologies between species with telencephalic deformations that result in differential axial bending, or the rotation of structures. Errors of interpretation can result from naive non-topologic neuromorphologic attributions. The ability to maintain consistent naming conventions between species greatly aids comparative studies and has been vital to the investigation of the functional role of many structures. There is a particular need for a naming system that captures true topological homologs across vertebrate groups, even when developmental axes have been deformed. Richly illustrated atlases of the developing human brain, such as those of Hochstetter (1929), Feess-Higgins and Larroche (1987) and Bayer and Altman (2008), can be of great assistance in this endeavor.

## Conclusion

An understanding of evolutionarily conserved pallial topology, combined with an appreciation of the variable morphogenetic deformations of the hemisphere that occur after dorsal pallial expansion, can guide the interpretation of homologous nuclei between species. The claustrum, like many other telencephalic structures, is forced to adopt a deformed C shape along the septo-amygdaloid axis in primates and thus requires careful interpretation in coronal sections. An understanding of these conserved topological relationships, alongside differential gene expression, has guided our identification of the claustral components in primates. The concept of a core and shell region in the ventral claustrum, and the existence of a distinct dorsal claustrum appear to be supported by current data in both rodents and primates. As anticipated, the dorsal endopiriform nucleus is only found deep to the piriform cortex and periamygdaloid olfactory cortex in the macaque. These configurations will require further validation using sections that are orthogonal to the topological caudal part of the claustral long axis in primates. Understanding the deformations that impact claustral topology will provide a framework for future studies and aid the interpretation of topographic organization within the claustrum.

## Data Availability

Publicly available datasets were analyzed in this study. This data can be found here: blueprintnhpatlas.org, mouse.brain-map.org.

## Author Contributions

The primary authors were DB and LP. CW contributed to the writing and editing of the article.

## Conflict of Interest Statement

The authors declare that the research was conducted in the absence of any commercial or financial relationships that could be construed as a potential conflict of interest.
